# Amputation Following Hand Escharotomy in Patients with Burn Injury

**Published:** 2016-03-02

**Authors:** Scott M. Schulze, Dexter Weeks, Joshua Choo, Damon Cooney, Alyssa L. Moore, Matt Sebens, Michael W. Neumeister, Bradon J. Wilhelmi

**Affiliations:** ^a^Division of Plastic Surgery, Department of Surgery, Southern Illinois University School of Medicine, Springfield; ^b^University of Louisville School of Medicine, Louisville, Ky; ^c^Division of Plastic Surgery, Department of Surgery, University of Louisville School of Medicine, Louisville, Ky

**Keywords:** hand escharotomy, hand burn amputation, burn patient escharotomy, amputation burn patient, hand burn

## Abstract

**Objective:** Hand burns are commonly seen in patients with burn injury. In the past, focus was on lifesaving measures, but with advances in burn care during the last century, the paradigm shifted to digital salvage and eventually to functional digital salvage. Good outcomes are heavily dependent on the care that is rendered during the initial management of the burn. **Methods:** A retrospective medical record review was conducted through the Central Illinois Regional Burn Center Patient Registry. Patients with burn injury treated with upper extremity and hand escharotomy between January 1, 2000, and December 31, 2005, were included in the study. **Results:** We identified a total of 34 patients with 57 burned hands. Six hands required delayed amputation of digits despite recognition of neurovascular compromise and escharotomy, yielding a 10% amputation rate. No correlation could be drawn with regard to total body surface area, age, or sex. **Conclusion:** Important principles in the acute phase include early splinting, recognition of the need for escharotomy and complete escharotomy when necessary, early excision and grafting, and involvement of occupational therapy for splinting and to guide both active and passive exercises. Although uncommon, some extremity burns may require subsequent amputation despite prompt attention and optimal treatment. In our case series, the need for amputation after successful escharotomies of salvageable digits was associated with full-thickness and electrical burns.

Burns of the hand are a common occurrence. It is estimated that hands are involved in more than 80% of all severe burns.^1^ A study conducted by the US Army Institute of Surgical Research found that 88.6% of 568 patients with burn injury accrued in a 2-year period had burns of the upper extremity.^2^ This high rate is often attributed to a protective reflex where patients shield their faces with their hands. Burn and scald injuries lead the way in workplace injuries that result in missing an average of 5 days per year per burn from work.^3^ Burns of the hand can have disastrous outcomes if aggressive intervention is not initiated early.

Even small burns to the hand can result in disfigurement and loss of function. Hand burns are more difficult to conceal than those to the extremities and trunk. Loss of the ability to perform activities of daily living is life altering, and preservation of activities of daily living has a large impact. Sheridan and associates^4^ stratified 1047 patients who had acutely burned hands. They demonstrated that 90% of patients had their activities of daily living restored with proper management of burns to the extensor mechanism, joint capsule, or bone.

Good outcomes depend on the care rendered during initial burn management. We feel that recognizing the need for escharotomy is the most overlooked. Prior studies have shown that 21% of digits develop necrosis and require amputation without escharotomy in hand burn injuries.^2^ With prompt intervention, 97% of patients with superficial injuries and 81% with deep dermal injuries can have normal hand function.^4^

## METHODS

A retrospective medical record review was conducted through the Central Illinois Regional Burn Center Patient Registry. Patients with burn injury treated with upper extremity and hand escharotomy between January 1, 2000, and December 31, 2005, were included in the study. Outcomes and demographics were compiled and analyzed.

## RESULTS

Thirty-four patients with 57 burned hands were identified. The average total body surface area was 50%. Coexisting inhalational injury was seen in 59% of the patients, and 35% of these patients died as a result of their burns. There was a male predominance, with a male to female ratio of 3:1. The etiology of burns in this series was predominantly thermal, with 29 flame and 2 scald burns. Two additional patients had chemical burns and 1 patient had an electrical burn (Table 1). Six hands required amputation of 1 or more digits, resulting in a 10% amputation rate for this series (Table 2). In each case, restoration of perfusion as measured by Doppler signals was achieved following escharotomy. The amputations ranged from postburn day (PBD) 10 to PBD 22. No correlation could be drawn between the need for amputation and total body surface area, age, or sex. The patients who required amputation after escharotomy had either full-thickness (FT) burns or electrical burns.

The 5 hands requiring amputation following escharotomy are summarized as follows:

Patient 1 was a 37-year-old man who sustained FT circumferential thermal burns to all fingers bilaterally. He underwent radial and ulnar-sided escharotomies to the hands and fingers at admission. On PBD 15, all fingers were necrotic and he was taken to the operating room (OR). All fingers of the left hand were amputated to the metacarpophalangeal (MCP) joint. All fingers of the right hand were amputated just proximal to proximal interphalangeal (PIP) joint. The patient survived.
Patient 2 was a 29-year-old man who sustained an FT circumferential thermal burn to the right hand and the small finger (SF). The patient also had significant damage to the ulnar nerve at admission and underwent radial and ulnar-sided escharotomy of the right hand and SF. On PBD 10, the right SF was necrotic and deemed unsalvageable. The patient was taken to the OR for amputation of the right SF up to between the MCP and PIP joints. The patient survived.
Patient 3 was a 40-year-old woman who sustained FT circumferential thermal burn to her hands bilaterally. She underwent radial and ulnar-sided escharotomy of the bilateral hands and fingers at admission. On PBD 22, all fingers appeared necrotic and she was taken to the OR. Her right thumb and index finger and left thumb, index finger, and little finger were amputated through the metacarpals. Her right little finger, ring finger, and SF and her left ring finger and SF were amputated up to between the MCP and PIP joints. The patient eventually expired because of her burn injuries.

## DISCUSSION

Escharotomy for constricting burns was first described by Fabry of Hilden, the father of German surgery, in 1607. It was not until the 1950s, when Blocker and Moyer championed the use of decompressive escharotomy to relieve vascular compromise, that escharotomy saw widespread use.^5^ Salisbury and Levine^2^ later demonstrated the clinical efficacy of escharotomy by showing that it resulted in an almost 3-fold increase in the number of salvaged phalanges.

**Figure 1:**
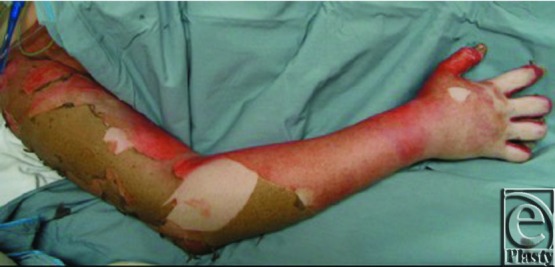
Burned extremity with features of neurovascular compromise.

Techniques for extremity escharotomy vary, but the principle is to maintain perfusion of the extremity while avoiding injury to vital underlying structures. Indications for escharotomy take into account the P's of neurovascular compromise: poikilothermia, pallor, paresthesia, and pulselessness (Fig 1). Absence of Doppler signals in the palmar arch, absence of capillary refill, and evidence of compartment syndrome are strong indicators for escharotomy. Pulselessness and absence of Doppler signals are late findings. The hand may take on a clawed position, also referred to as the intrinsic minus position (MCP joints in extension and PIP joints in flexion).

Escharotomy can be carried out safely at a prepared bedside. The authors prefer to use conscious sedation with hemodynamic monitoring. Small circumferential burns or situations in which conscious sedation is contraindicated may be managed by infiltration with a dilute local anesthetic such as 0.5% bupivacaine (Marcaine). The initial skin incision may be made with a scalpel or electrocautery; however, the surgeon must be prepared for brisk bleeding, which requires electrocautery for hemostasis.

The escharotomy should extend beyond the axial boundaries of the constricting eschar. Proper depth is not reached until the underlying fat bulges from the wound. The authors prefer to start with a radial incision that extends to the tip of the acromion proximally and to the lateral aspect of the antecubital flexion crease distally. Distal neurovascular compromise may be relieved with a proximal escharotomy above the elbow depending on the extent of the burn. Often the incision is continued down the radial aspect of the forearm to a point just posterior to the styloid process of the radius at the flexion crease of the wrist. The main nerve at risk with this approach is the superficial sensory branch of the radial nerve, which travels down the forearm deep to the brachioradialis but becomes superficial as it pierces the deep fascia near the radial aspect of the wrist.

**Figure 2:**
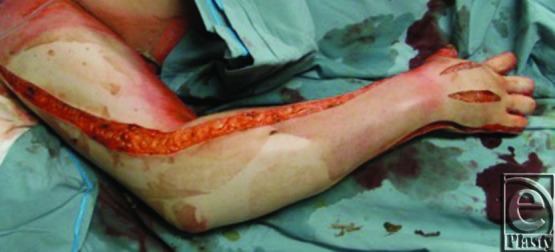
Burned extremity after escharotomy.

A separate ulnar incision is sometimes required, which courses from the axilla proximally to the medial aspect of the antecubital flexion crease distally. Frequent assessment of perfusion dictates the extent of the incision. The surgeon must avoid injuring the ulnar nerve and artery at the distal ulnar wrist when passing superficial to the flexor retinaculum into the hand. If blood flow has not been reestablished, both incisions may be carried onto the thenar and hypothenar eminences along the border of the glabrous and nonglabrous skin. If Doppler signals are absent in the fingers, hand and digital escharotomies may be necessary. The surgeon extends the radial incision dorsally onto the extensor surfaces of the hand and on to the fingers along the midaxillary line dorsal to the flexion creases. Several variations exist as to the placement of these incisions. Consideration should be given to the potential need for intrinsic muscle decompression of the hand (which involves dorsal incisions over the second and fourth metacarpals), avoiding injury to the extensor tendons, and opposition sides of the digits. When carried to the digits, the authors prefer to place the incisions along the ulnar aspect of the index finger, little finger, and ring finger and the radial aspect of the thumb and SF. The radial and ulnar sides of all fingers are incised if there is residual neurovascular compromise (Fig 2).

Electrical burns commonly present persistent vascular compromise requiring fasciotomy of the forearm and hand compartments. Utilizing the prior escharotomy incision is ideal. The radial escharotomy in the forearm facilitates release of extensor and mobile wad compartments; release of the volar compartment, however, usually requires a separate volar incision that allows assessment of the deep flexor compartment. If a separate ulnar escharotomy was already performed, the superficial and deep volar compartments are safely assessed between the flexor carpi ulnaris and the flexor digitorum superficialis.

**Figure 3:**
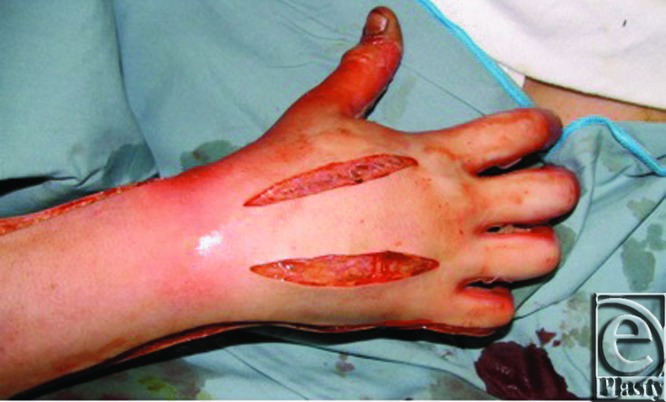
Decompression of the dorsal interossei.

The dorsal interossei may need decompression of significant edema. This is conducted through 2 axial incisions along the radial aspect of the second metacarpal and the ulnar aspect of the fourth metacarpal, allowing access to all volar and dorsal interosseous compartments and the adductor compartment of the thumb while minimizing extensor tendon exposure (Fig 3). Hemostasis is achieved, and the extremities are supported above the level of the heart to induce gravity drainage.

Cope and Moore, and later Baxter, found that although the most rapid edematous accumulation occurs within the first few hours after a thermal burn, edema was maximum between 18 and 36 hours.^6^ Therefore, if prophylactic escharotomy is not done, frequent reassessment of neurovascular status is done. The utility of prophylactic escharotomy was detailed by Robert Carlotto and based on the following criteria: (1) there are circumferential or near-circumferential burns that are clearly deep partial thickness or FT, (2) a large fluid volume resuscitation is expected on the basis of the extent of the associated burn injury, and (3) serial reassessment of the hand may be difficult because of diminished level of consciousness, systemic hypothermia, or hypotension.^7^

Various studies detail blood flow quantification to determine adequate perfusion.^5^^,^^6^^,^^8^^,^^9^ These include the use of Doppler flowmeters, Xenon-133 washout determinations to assess muscle blood flow, and infrared photoplethysmography. The use of Doppler examination in conjunction with serial examinations has the best yield in experienced hands. It is important to realize that if Doppler signals are lost, there may be a lag in return of these signals after successful intervention. Moylan and associates^5^ noted, when using the Doppler flowmeter, it took up to 24 hours for *return* of signals after upper extremity escharotomies. This shows that the absence of Doppler signals immediately after escharotomy does not imply nonviability.

Doppler examinations should be done overlying the palmar arch and the digital arteries. Completely normal ulnar or radial artery Doppler examination does not equate to hand perfusion, just as good palmar signals do not equate to appropriate digital perfusion. The digits lack *venae comitantes* accompanying the digital arteries. Therefore, the fingers drain via other deep low-pressure veins and superficial dorsal veins. Since dorsal hand burns far outnumber palmar burns, secondary to clenching of the hand during protective mechanisms, these superficial veins are often damaged. As edema and circumferential burn eschar decrease digital outflow, edema and future perturbation of blood flow dramatically diminish arterial flow. This is where clinical judgment outweighs any direct or indirect method of determining flow.

Splinting of the effected hand and digits is tantamount to acute burn treatment. The burned hand may take on a tense, edematous claw-like position as the joints relax. Proper splinting is mandatory to ensure the tendons are maximally stretched. This “safe” position places the wrist at 20° to 30° of extension (at least 10° less than maximum), the MCP joints are flexed at 60° to 70° and the interphalangeal joints are fully extended.^10^ The thumb is splinted in abduction to maintain the web space. This intrinsic plus position allows for much less stiffness than if the hand was splinted otherwise. Until being taken for excision and grafting, patients receive ranging of the hand and finger joints twice daily. After excision and grafting, the hand is kept in occlusive dressing for 3 to 5 days’ duration. After removal of the dressings to assess graft take, it is essential to start active range of motion exercises with a dedicated therapy team.

There is significant difference between a salvaged digit and a functional salvaged digit. This must be discussed with the patient before attempting salvage or amputation. The surgeon and the patient must recognize that deep partial-thickness and FT burns, especially those characterized as fourth degree, are at risk for necrosis despite appropriate and timely treatment. In our experience, digital amputation following escharotomy is found with increasing frequency in deep thermal burns. In electrical injuries, the tissue damage often extends deeper than can be superficially inspected. Other theories to explain the progressive necrosis suggest small vessel occlusion and elevated levels of arachidonic acid in areas of greatest heat production. The latter has been demonstrated in animal studies, as antithromboxane agents halt the progression of necrosis.^11^ With regard to deep thermal burns, direct damage to the digital arteries leads to the necrosis requiring amputation.

Many authors have reported high-voltage injury requiring amputation despite early intervention.^11^^-^13 Indications for amputation in the setting of thermal injury have been only sparsely reported in the literature.

## CONCLUSION

Treatment of the burned hand requires aggressive early management to optimize functional outcomes. When formulating a treatment plan, the clinician must take into account the neurovascular status of the extremity, proper fluid resuscitation, and clinical course established by serial observations. If there is a circumferential or near-circumferential burn that impedes blood flow, escharotomies are carried out prior to the presentation of late findings, such as lack of distal Doppler signals. Splinting in the safe position and elevation is essential to avoid stiffness and minimize edema. After successful demonstration of graft survival, occupational therapy with active range of motion is initiated to return function.

Even with timely and proper management, limb and digit loss can still occur. The most important factor related to eventual need for amputation is the extent of damage at the initial insult. Full-thickness circumferential burns and electrical injuries may require eventual amputation despite optimal management, even if they may appear salvageable initially.

## Figures and Tables

**Table 1:** Etiology of burns

Etiology	Number of patients	Percentage of patients
Flame	29	85
Scald	2	6
Chemical	2	6
Electrical	1	3

**Table 2:** Patients requiring amputation*

	Sex	Age	Etiology	Depth	PBD	Digits amputated	Patient outcome
1	M	37	Thermal	FT	15	**L**—All fingers up to MCP **R**—All fingers up to between MCP and PIP	S
2	M	29	Thermal	FT	10	**R**—SF up to between MCP and PIP	S
3	F	40	Thermal	FT	22	**L**—Thumb, IF, and LF through metacarpals and RF and SF up to between MCP and PIP **R**—Thumb and IF through metacarpals and LF, RF, and SF up to between MCP and PIP	E

* PBD indicates postburn day; L, left; MCP, metacarpophalangeal; R, right; PIP, proximal interphalangeal; SF, small finger; IF, index finger; LF, little finger; and RF, ring finger.

## References

[1] Germann G, Philipp K, Green DP (2005). The burned hand. Greens Operative Hand Surgery.

[2] Salisbury RE, Levine NS (1976). The early management of upper extremity thermal injury. Major Probl Clin Surg.

[3] National Institute for Occupational Safety and Health (2004). Worker Health Chartbook 2004.

[4] Sheridan RL, Hurley J, Smith MA (1995). The acutely burned hand: management and outcome based on a ten-year experience with 1047 acute hand burns. J Trauma.

[5] Moylan JA, Wellford WI, Pruitt BA (1971). Circulatory changes following circumferential extremity burns evaluated by the ultrasonic flowmeter: an analysis of 60 thermally injured limbs. J Trauma.

[6] Clayton JM, Russel HE, Hartford CE, Boyd WC, Barnes RW (1977). Sequential circulatory changes in the circumferentially burned limb. Ann Surg.

[7] Carlotto R (2005). The burned hand: optimizing long-term outcomes with a standardized approach to acute and subacute care. Clin Plast Surg.

[8] Salisbury RE, Taylor JW, Levine NS (1976). Evaluation of digital echarotomy in burned hands. Plast Reconstr Surg.

[9] Smith DR Jr, Benedick PJ, Madison SA (1984). Evaluation of vascular compromise in the injured extremity: a photoplethysmographic technique. J Hand Surg [Am].

[10] Gillespie JN, Clarke MS, Wilhelmi BJ Intrinsic plus hand. eMedicine.

[11] Bingham H (1986). Electrical burns. Clin Plast Surg.

[12] Rai J, Jeschke M, Barrow R (1999). Electrical injuries: a 30-year review. J Trauma.

[13] Hussmann J, Kucan J, Russell R (1995). Electrical injuries—morbidity, outcome and treatment rationale. Burns.

